# Supporting data on enhanced reprogramming of human CD34+ hematopoietic stem cells to induced pluripotent stem cells using human placenta-derived cell conditioned medium

**DOI:** 10.1016/j.dib.2020.106140

**Published:** 2020-08-06

**Authors:** Seung-Jin Lee, Ji-Hea Kim, Ka-Won Kang, Young Park, Byung-Soo Kim

**Affiliations:** a Institute of Stem Cell Research, Korea University College of Medicine, Seoul, South Korea; b Department of Biomedical and Science, Graduate School of Medicine, Korea University, Seoul, South Korea; c Department of Internal Medicine, Anam Hospital, Korea University Medical Center, Seoul, South Korea

**Keywords:** Reprogramming, Human induced pluripotent stem cells, Human placenta-derived cell conditioned medium, Hematopoietic stem cells, Acute lymphoblastic leukemia

## Abstract

The data presented herein support “Generation of an induced pluripotent stem cell line KUMCi001-A from CD34+ bone marrow cells of a patient with acute lymphoblastic leukemia using human placenta-derived cell conditioned medium.” The supplementary data were as follows. (1) Comparison of reprogramming efficiency of human placenta-derived cell conditioned medium with defined medium (mTeSR™1) and the generation of induced pluripotent stem cells (iPSCs) from a patient with acute lymphoblastic leukemia (ALL) with significantly higher reprogramming efficiency than that of the defined medium (*P* ≤ 0.05). (2) Evaluation of differentiation capability of the generated ALL_iPSCs into hematopoietic stem cells (HSCs) and comparison with normal iPSCs using the colony-forming unit (CFU) assay. ALL_iPSCs manifested all lineages for hematopoiesis in their colonies similar to normal iPSCs. (3) ALL_iPSCs showed a considerably higher number of burst-forming unit-erythroid colonies indicating the presence of more erythroid progenitors than normal iPSCs; this tendency was confirmed in the CFU assay of ALL_CD34+ cells. This has been previously reported as a feature of ALL. Thus, the hematopoietic characteristics of the donor patient with ALL appear to be maintained in our ALL_hiPSC line although the karyotype was normalized during reprogramming.

**Specifications Table**

**Table:** 

**Subject**	Cell Biology
**Specific subject area**	Human induced pluripotent stem cells (hiPSCs)
**Type of data**	Graph Figure
**How data were acquired**	ALP staining (ES Cell Characterization Kit) CFU assay (MethoCult™ H4434 Classic) Data analysis (GraphPad Prism version 6.0) Image capture (IX71 microscope)
**Data format**	Raw Analyzed
**Parameters for data collection**	CD34+ cells were isolated from a patient with acute lymphoblastic leukemia and reprogrammed to iPSCs using an existing method (with mTeSR™1 medium on a matrigel-coated dish) and human placenta-derived cell conditioned medium (hPCCM). We evaluated whether ALL_iPSCs, similar to normal iPSCs, could generate hematopoietic stem cells (HSCs) through embryoid body formation and performed the colony-forming unit (CFU) assay.
**Description of data collection**	The reprogramming efficiency was measured by alkaline phosphatase (ALP) staining. The ALP-positive colonies were counted and quantified using Prism software. For hematopoietic cells, the CFU assay was performed with methylcellulose medium and hematopoietic cell clusters were enumerated based on morphology.
**Data source location**	Korea University Anam Hospital Seoul Republic of Korea
**Data accessibility**	All data are available with this article.
**Related research article**	Lee SJ et al. 2020 Generation of an induced pluripotent stem cell line KUMCi001-A from CD34+ bone marrow cells of a patient with acute lymphoblastic leukemia using human placenta-derived cell conditioned medium Stem Cell Research: Lab resource In Press.

**Value of the data**

• These data can help accelerate the reprogramming of human CD34+ cells to hiPSCs using hPCCM, study the mechanisms underlying the pathophysiology of ALL, and screen patient-specific drugs.
• This hiPSC line can be a promising model for research on acute leukemia and drug screening.
• These data can be used to optimize reprogramming of iPSCs from CD34+ cells of patients with leukemia in humanized conditions using hPCCM; blood cancer patient-specific experiments can be made possible by producing disease-specific hiPSC line.

## 1 Data description

**Fig. 1:**
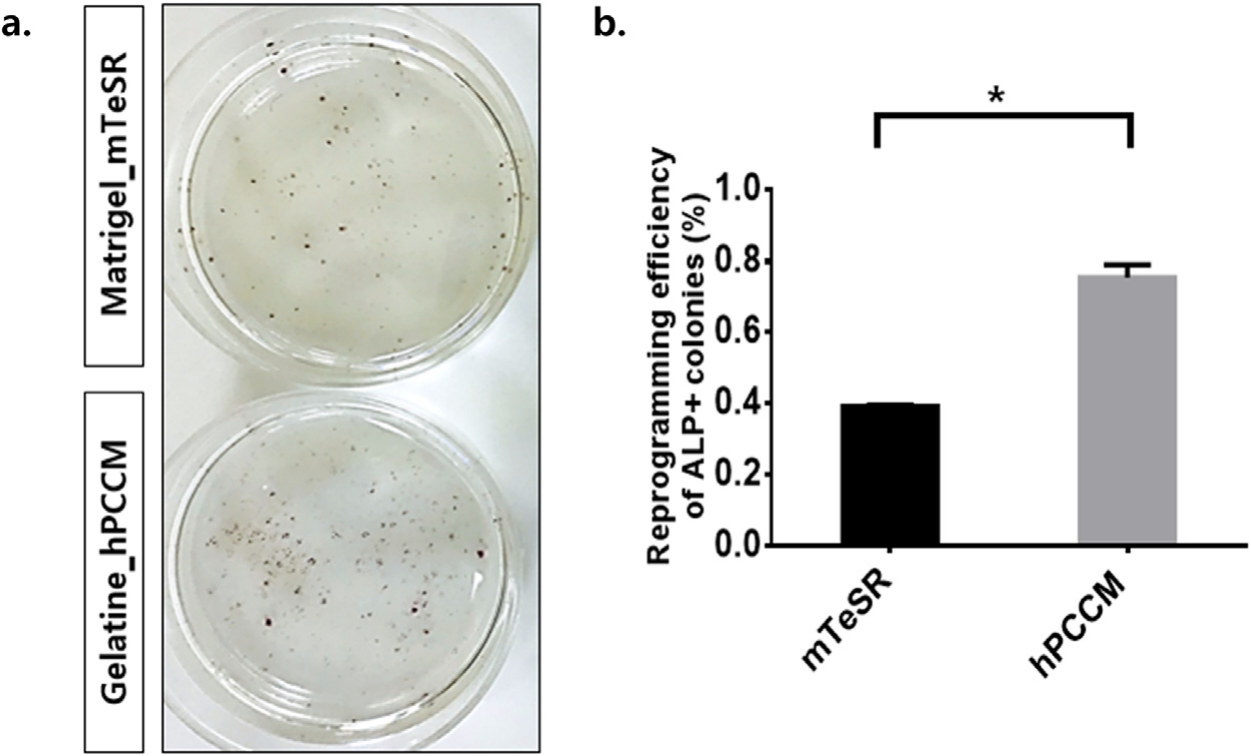
Generation of induced pluripotent stem cells (iPSCs) in human placenta-derived cell conditioned medium (hPCCM) from ALL CD34+ cells. (a) Alkaline phosphatase (ALP) staining of reprogrammed iPSCs. The reprogrammed colonies were assessed in the presence of mTeSR (upper plate) or hPCCM (lower plate). (b) The bar graph shows the percentage of ALP-positive colonies and reprogramming efficiency. Data are presented as mean ± SEM of three independent experiments, *P ≤ 0.05.

**Fig. 2:**
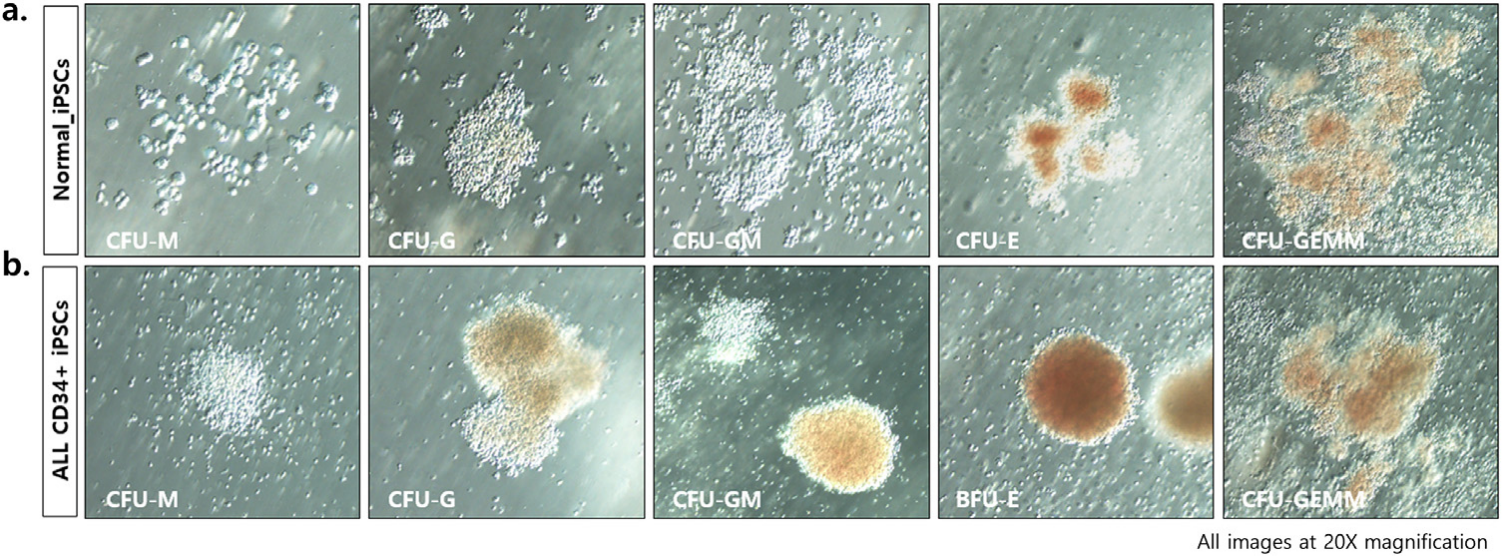
iPSC-derived hematopoietic stem cells (HSCs) produce colonies of multiple cell lineages, and they were assessed using the colony-forming unit (CFU) assay with methylcellulose-based medium. (a) Normal iPSC-derived HSCs and (b) ALL CD34+ iPSCs-derived HSCs. The hematopoietic cell types observed included granulocyte/macrophage (CFU-M, CFU-G, and CFU-GM), erythroid (BFU-E and CFU-E), and mixed (CFU-GEMM) colonies.

**Fig. 3:**
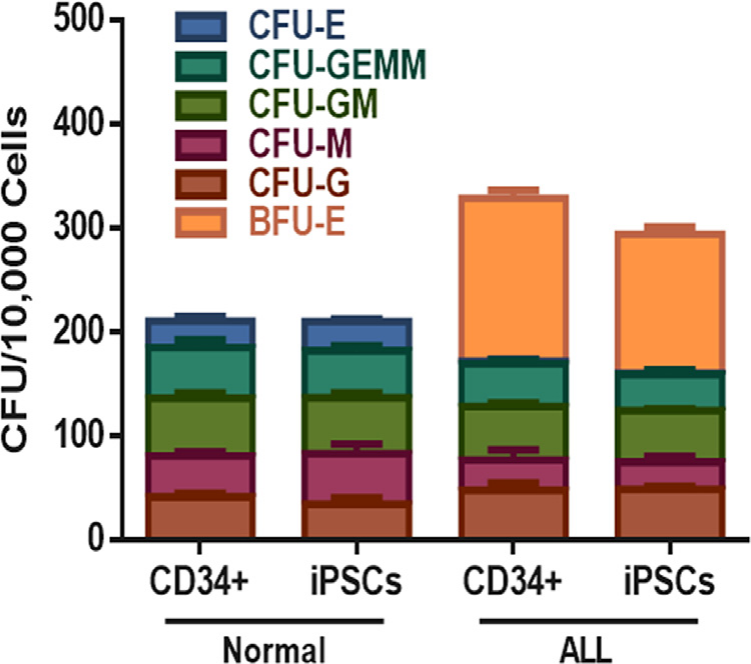
Number of CFU observed in cultures of HSC derived from normal iPSCs and ALL_iPSCs or normal CD34+ and ALL CD34+ cells. iPSCs and CD34+ from the patient with ALL, and the number of BFU-E colonies was significantly higher than the normal level. Data are presented as mean ± SEM of three independent experiments.

Based on the findings of our previous study [1], we compared the efficiency of reprogramming human patient cells using the human placenta-derived cell conditioned medium (hPCCM) protocol and commonly recognized reprogramming protocols (mTeSR) by alkaline phosphatase (ALP) staining. The reprogramming efficiency of cells cultured in hPCCM was higher than that of cells in mTeSR (Fig. 1 and Table 1). The generated iPSCs were further compared with normal iPSCs using the CFU analysis to determine whether they had the ability to differentiate into HSCs (Fig. 2). Moreover, ALL_iPSCs showed a higher number of burst-forming unit-erythroid (BFU-E) colonies than normal iPSCs. In addition, the origin cell of iPSCs, CD34+, showed the same pattern and maintained the characteristics of ALL in the CFU assay (Fig. 3). We have demonstrated that the production of BFU-E is significantly increased in patients with ALL, both CD34+ cells and differentiated HSCs from iPSCs, using the CFU assay (Table 2).

**Table 1:** Reprogramming efficiency of ALP positive colonies (%).

Condition	Cells plated	Colonies/well	Efficiency (%)
mTeSR	1 × 10^5^	392 ± 0.008	0.392
hPCCM	1 × 10^5^	684 ± 0.754	0.445

*P ≤ 0.05.

**Table 2:** Number of colony forming units (*n* = 3).

Colony types	Normal		ALL_ Patient	
	CD34+	iPSCs	CD34+	iPSCs
CFU-G	41.3 ± 3.21	34 ± 6.5	47.6 ± 6.8	48 ± 3
CFU-M	39.3 ± 4	49 ± 9	29.3 ± 9.5	26.3 ± 5.5
CFU-GM	55.6 ± 5	53.6 ± 4.7	51 ± 3.6	49.6 ± 1.5
CFU-CEMM	48.6 ± 7.7	45.3 ± 4.5	41.3 ± 3	34.6 ± 4.5
CFU-E	25.3 ± 4.5	28±2.6	2.6 ± 2	1.3 ± 0.5
BFU-E	ND	ND	156 ± 7.6	133 ± 7.2

ND, Not Determined.

Table 1. Alkaline phosphatase (ALP) staining of the reprogrammed iPSCs 7 days after transduction; then, ALP-positive colonies were counted. Data are presented as mean ± SEM of three independent experiments.

Table 2. CFU assay of CD34+ cells or differentiated HSCs from iPSCs of healthy individuals or patients with ALL. Number of colonies per 10,000 cells. Data represent the mean of three independent experiments ± SEM.

## 2 Experimental design, materials and methods

### 2.1 Isolation of CD34+ cells

The bone marrow was collected from a patient with ALL, and mononuclear cells (MNCs) were separated using Ficoll-Paque™ Plus medium (GE Healthcare Life Sciences, Seoul, South Korea). We separated CD34+ cells from MNCs by labeling with CD34 magnetic beads (Miltenyi Biotec, BergischGladbach, Germany) according to manufacturer's instructions and collected them. We determined the purity of the sorted CD34+ cells (over 90%) by flow cytometry. Antibodies against anti-human CD34-FITC (Becton Dickinson, CA, USA) were used at 1:100 dilution and analyzed using FACSCalibur™ (Becton Dickinson, CA, USA).

### 2.2 Cell culture

Normal CD34+ cells were purchased from Lonza Bioscience (Walkersville, MD, USA) and cultured in Stemline II Hematopoietic Stem Cell Expansion Medium (Sigma Aldrich, St. Louis, USA) containing 100 ng/mL stem cell factor (SCF), thrombopoietin (TPO), and G-CSF (R&D Systems, MN, USA). The medium was changed every 2 days. Normal bone marrow-derived human iPSCs (IISH1i-BM1) were purchased from the WiCell Research Institute (Madison, WI, USA) and handled in accordance with the manufacturer's instructions, and the medium was changed daily. All cells were cultured at 37 °C in 5% CO_2_.

### 2.3 Generation of iPSCs

CD34+ cells from the bone marrow of a patient with ALL were generated using the CytoTune-iPS 2.0 Sendai Reprogramming Kit (Life Technologies, Waltham, MA, USA), per the manufacturer's instructions and as previously described [1]. Briefly, CD34+ cells were transduced with Sendai viruses at MOI = 5 in Stemline II Hematopoietic Stem Cell Expansion Medium with 100 ng/mL SCF, 100 ng/mL TPO, and 50 ng/mL G-CSF. The day after transduction, the cells (1 × 10^5^) were transferred onto a 0.1% gelatin-coated 35-mm dish in hPCCM or matrigel-coated 35-mm dish in mTeSR. On the subsequent day, half the medium was replaced with hPCCM or mTeSR, respectively, and the entire medium was changed every day until day 7 and colony picking.

### 2.4 Alkaline phosphatase (ALP) staining

To confirm pluripotency, the cells were stained with ALP. ALP activity was detected using the ES Cell Characterization Kit (Millipore, Burlington, MA, USA) in accordance with the manufacturer's instructions, and by manually counting positive colonies.

### 2.5 Differentiation of hematopoietic cells

The undifferentiated hiPSCs were cultured in low-adherence surface plates with DMEM-F12 medium, containing 20% knockout serum replacement for 7 days. Subsequently, they were incubated with 5 ng/mL bone morphogenetic protein 4 (BMP4) for the first 3 days and in Stemline II serum-free medium (Sigma) for 7 days. This medium was supplemented with 20 ng/ml vascular endothelial growth factor (VEGF), basic fibroblast growth factor (bFGF), stem cell factor (SCF), FMS-like tyrosine kinase 3 (Flt3), and thrombopoietin (TPO) (R&D Systems, Minneapolis, USA).

### 2.6 CFU assay

The CFU assay was performed with 10,000 differentiated hematopoietic cells or CD34+ cells seeded in methylcellulose medium (Stem Cell Technologies, Vancouver, Canada). After incubation for 14 days, at 37 °C in 5% CO_2_, hematopoietic cell clusters were enumerated based on morphology.

### 2.7 Identification of CFU morphology

We evaluated HSC frequency by colony size and cellular composition. The most primitive CFU that can be measured using morphological and phenotypic criteria contains granulocytes, erythrocytes, macrophages, and often megakaryocytes; an explanation of colony types is provided below.

*Colony forming unit-granulocyte (CFU-G)*: A colony containing a homogeneous population of eosinophils, basophils, or neutrophils. Large colonies have one or more dense dark cores.

*Colony forming unit-macrophage (CFU-M)*: A colony containing a homogenous population of macrophages. Individual cells can usually be distinguished, particularly at the edge of the colony.

*Colony forming unit-granulocyte, macrophage (CFU-GM)*: A colony containing a heterogeneous population of macrophages and/or granulocytes. It does not appear hemoglobinized (no red or brown). The morphology is similar to that of CFU-M and CFU-G.

*Colony forming unit-erythroid (CFU-E)*: A colony containing approximately 200 hemoglobinized erythroblasts in only one or two small clusters. It appears red or brown.

*Burst forming unit-erythroid (BFU-E)*: A colony containing small (3–8 clusters), intermediate (9–16 clusters), or large (>16 clusters) according to the number of clusters present. The cells are hemoglobinized (red or brown) and are difficult to distinguish individual cells within each cluster.

*Colony forming unit-granulocyte, erythrocyte, macrophage, megakaryocyte (CFU-GEMM)*: A colony containing multi-lineage cells that give rise to erythroid, granulocyte, macrophage, and/or megakaryocyte lineages. It can have both erythroid (hemoglobinized) cells and 20 or more non-erythroid (not hemoglobinized) cells. It is usually larger than the CFU-GM or BFU-E colonies.

### 2.8 Statistical analyses

Statistical significance was evaluated using the Student's *t*-test for unpaired analysis. All experiments were performed in triplicates. Results with a *P* value of <0.05 were considered statistically significant. Statistical analyses were carried out using GraphPad Prism 6 (GraphPad Software, Inc., La Jolla, CA).

## Ethics statement

The study was approved by the Institutional Review Board of Anam Hospital (IRB#2020AN0067) at the Korea University Medical Center. The bone marrow was harvested in a clean germ-free facility at the Korea University Anam Hospital. Informed consent was obtained from the donor, in accordance with the tenets of the Declaration of Helsinki.

## Declaration of Competing Interest

The authors declare that they have no known competing financial interests or personal relationships, which have, or could be perceived to have, influenced the work reported in this article.

## Appendix Supplementary materials

Image, image 1
