# Supplementary figures and images for: Supporting data on enhanced reprogramming of human CD34+ hematopoietic stem cells to induced pluripotent stem cells using human placenta-derived cell conditioned medium
Source: Data Brief. 2020 Aug 6;32:106140. doi: 10.1016/j.dib.2020.106140 (PMC7452686; doi:10.1016/j.dib.2020.106140)

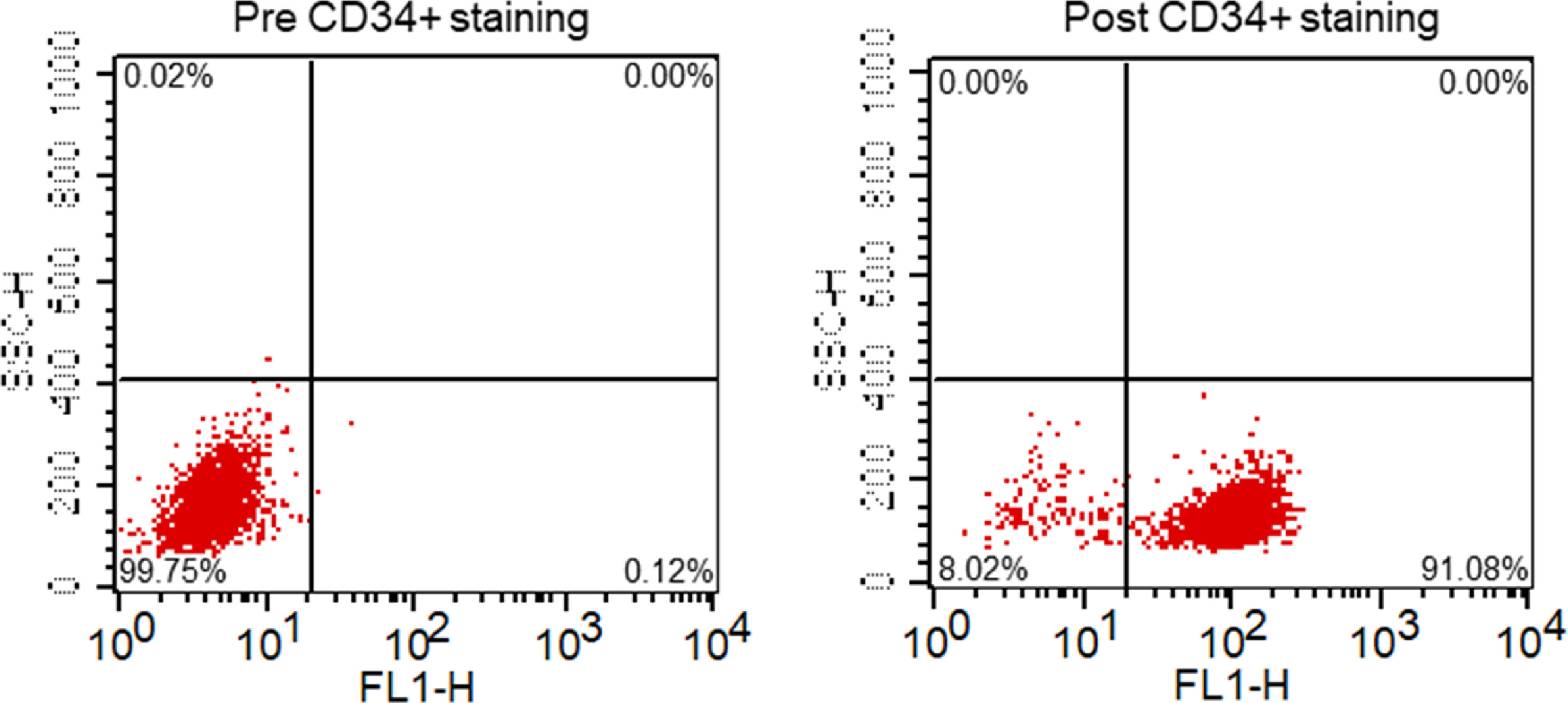

Supplement: Supplementary file 1 [file mmc1.jpg]
